# Establishment of malignantly transformed dendritic cell line SU3-ihDCTC induced by Glioma stem cells and study on its sensitivity to resveratrol

**DOI:** 10.1186/s12865-018-0246-z

**Published:** 2018-02-02

**Authors:** Xifeng Fei, Anqi Wang, Delin Wang, Xan Meng, Jiawei Ma, Lei Hong, Ruwei Qin, Aidong Wang, Jun Dong, Qiang Huang, Zhimin Wang

**Affiliations:** 1 Department of neurosurgery, Suzhou Kowloon Hospital, Shanghai Jiaotong University School of Medcine, Wan’sheng Road 118, Suzhou, 215006 China; 2 Department of the Soochow University, Suzhou, 215004 China; 3 Department of the Second Affiliated Hospital of Soochow University, Suzhou, 215004 China

**Keywords:** Cell malignant transformation, Tumor stem cells, Tumor microenvironment, Tumor cell drug resistance, Resveratrol, Cisplatin

## Abstract

**Background:**

As a factor contributing to the tumor cell drug resistance, tumor microenvironment (TME) is being paid increasingly attention. However, the drug resistance of malignantly transformed cells in TME has rarely been revealed. This paper is designed to investigate the sensitivity of malignantly transformed cell line (ihDCTC) induced by glioma stem cells (GSCs) in TME to chemotherapeutic drugs.

**Methods:**

(1) Establishment of ihDCTC cell line,The bone marrow cells from enhanced green fluorescent protein (EGFP) transgenic nude mice were employed to culture the dendritic cells (DCs) in vitro, which were then co-cultured with red fluorescence protein (RFP) transgenic GSCs (SU3) to obtain ihDCTC (2) Res and Cis were used to intervene in the growth of abovemetioned cell lines in vitro and Res treated in bearing ihDCTC tumor mice, followed by evaluating their drug sensitivity and changes in key signaling proteins via half maximal inhibitory concentration (IC_50_), tumor mass and immunostaining method.

**Results:**

(1) ihDCTC could express CD11c and CD80 as well as possessed immortalized potential, heteroploid chromosomes and high tumorigenicity in nude mice in vivo. (2) At 24 h, 48 h and 72 h, the IC_50_ value of ihDCTC treated with Cis was 3.62, 3.25 and 2.10 times higher than that of SU3, while the IC_50_ value of ihDCTC treated with Res was 0.03, 0.47 and 1.19 times as much as that of SU3; (3) The xenograft mass (g) in vivo in the control, Res, Cis and Res + Cis groups were 1.44 ± 0.19, 0.45 ± 0.12, 0.94 ± 0.80 and 0.68 ± 0.35(x ± s) respectively. The expression levels of IL-6, p-STAT3 and NF-κB proteins in the xenograft tissue were significantly reduced only in the Res treatment group.

**Conclusion:**

In vitro co-culture with GSC can induce the malignant transformation of bone marrow derived dendritic cells, on the one hand, ihDCTC shows higher drug resistance to the traditional chemotherapeutic drug Cis than GSCs, but, on the other hand, appears to be more sensitive to Res than GSCs. Therefore, our findings provide a broader vision not only for the further study on the correlation between TME and tumor drug resistance but also for the exploration of Res anti-cancer value.

## Background

In recent years, one of the breakthroughs in the field of tumor immunology is the identification of the important role played by nonresolving inflammation (NRI) and myeloid-derived suppressor cells (MDSCs) in the tumor occurrence and development [1, 2]. Our research group has successfully established multiple malignant transformed cell lines by transplanting glioma stem/progenitor cells to the subcutaneous tissue, cranial cavity, abdomen, liver and other parts of EGFP transgenic nude mice [3, 4]. In the area of TME, the research on malignant transformation of non-tumor cells has formed a solid foundation. Tumor-associated dendritic cells (DCs) are an important member of MDSCs in TME. The malignant DCs used in our study is highly proliferative and prepared based on human GSC line SU3 (SU3-induced host Dendritic Cells Transformed Cell, ihDCTC). It is known that TME cells, especially fibroblasts, may facilitate tumor cell resistance to chemotherapeutic drugs in traditional tumor chemotherapy [5]. Furthermore, the action of various inflammatory factors and chemokines in the complicated TME may also disable the immune cells to kill the tumor cells, thereby maintaining tumor angiogenesis and aggravating tumor invasion and metastasis [6]. For example, tumor-associated macrophages (TAMs) are involved in tumor angiogenesis, matrix remodeling, invasion and metastasis, immunosuppression, and chemical resistance [7]. However, it remains unclear about the sensitivity of transformed cells in TME,such as ihDCTC, to chemotherapeutic drugs. This paper reported that ihDCTC is not sensitive to the traditional chemotherapeutic drug cisplatin (Cis) but sensitive to resveratrol (Res) and is regulated by IL-6/p-STAT3/NF-κB.

## Methods

### Materials

SU3 cells and experimental animals were prepared by our research group, SU3 cells were first obtained from the department of the Second Affiliated Hospital of Soochow University. The method of obtaining the cells is previously described [8], 4-6-week-old male and female GFP nude mice and non-fluorescent nude mice at an average weight of 22 g were provided by the Center for Experimental Animals, Soochow University (certificate No. SY X K (Su) 2007-0035) [9]. All the animals were bred and maintained in the Specific Pathogen Free Animal Care Facility,Nasal1000 grade. The RFP lentiviral vector was purchased from Shanghai Innovation Biotechnology Co., Ltd.; hamster anti-mouse CD11c antibody from eBioscience Corporation, US; APC-labeled anti-mouse CD11c antibody and APC-labeled anti-mouse CD80 antibody from Biolegend Corporation, US; recombinant mouse granulocyte-macrophage colony-stimulating factor (rmGM-CSF) and recombinant mouse interleukin 4 (rmIL-4) from Peprotech Corporation; rabbit anti-mouse α signal protein (SIRP-α) antibody from Abcam, Inc.; CCK-8 reagent from Dojindo Chemical Technology Co., Ltd.; immunohistochemical staining and Western Blot primary antibody reagents: antibodies against IL-6, STAT3, p-STAT3, NF-κB and p-NF-κB were purchased from Abcam, Inc. DMEM medium and fetal bovine serum were purchased from Hyclone Laboratories, Inc., US; flow cytometer from Beckman Coulter, US; fluorescent inverted microscope from Olympus Corporation, Japan; microplate reader from Tecan, Switzerland. Freezing microtome was purchased from LEICA, Germany and cell incubator from SANYO, Japan. Resveratrol and cisplatin were purchased from Gibco and Qilu Pharmaceutical Co., Ltd. Respectively.

### Establishment of SU3-ihDCTC cell line

#### Obtain immortalized DCs from co-cultured SU3-RFP and DCs

The mouse DC line was established and identified for corresponding molecular markers according to the classic Inaba method [10]. The established glioma stem/progenitor cell line SU3 was transfected with RFP gene (SU3-RFP) according to the manufacturer’s instructions, followed by puromycin resistance screening to obtain stable cell strain SU3-RFP; the strain was then placed to DMEM/F12 stem cell culture medium that contained B27 additive, 20 ng/ml epidermal growth factor (EGF) and 20 ng/ml recombinant human basic fibroblast growth factor (bFGF) for balling, followed by digesting into single cells; the above obtained SU3-RFP and DCs were directly mixed and cultured in DMEM/high glucose medium containing 10% fetal bovine serum subject to the ratio of 1:10; the growth status of co-cultured cells was observed under the fluorescence microscope every day until EGFP + cells became clustered or colonized, followed by 0.25% trypsin digestion and single-cell suspension collection via centrifugation; in the subculture process, single cells expressing EGFP rather than RFP were screened out under the fluorescence microscope and then separately passaged in 96-well plates, where 1 strain of EGFP positive cells that could be infinitely passaged, named SU3-ihDCTC, was selected for further analysis on their cytobiological characteristics; after that, amplification and passaging were conducted.

### Detection of the genetic characteristics of SU3-ihDCTC

RT-PCR and immunocytochemical staining were used to detect murine markers β-actin and EGFP in SU3-ihDCTC cells, DC molecular marker proteins D11c, CD80 [11] and SIRP-α [12], as well as mouse macrophage marker F4/80 [13]. The specific steps were as follows:

RT-PCR detection. ihDCTC cells in the logarithmic growth period were selected for the test. The total RNA of the collected and treated cells were extracted using Trizol reagent, followed by reverse transcription to synthesize cDNA according to the reverse transcription kit instructions. PCR reaction was conducted under the following conditions: predegeneration at 94 °C for 5 min, 30 cycles of denaturation at 94 °C for 30s and renaturation at 72 °C for 30s, and extension at 72 °C for 7 min. β-actin was taken as the internal reference. PCR products were processed on the 1.5% agarose gel electrophoresis (AGE) at 100 V for 30 min, and the gel image processing system was used for imaging; the primers were synthesized by Suzhou Genewiz Co., Ltd. (Table 1).

**Table 1:** Primer name, sequence and product length

Primer name	Primer sequence(5′-3′)	Product length
EGFP	F:GCCACAAGTTCAGCGTGTCCG R:GTTGGGGTCTTTGCTCAGGGCG	566 bp
RFP	F:AGGTTCTTAGCGGGTTTCTTG R:CTTCCCTGAGGGCTTCACAT	312 bp
CD68	F:CTACATGGCGGTGGAATACAATG R:TAGCCTTAGAGAGAGCAGGTCAA	175 bp
F4/80	F:CAGCTGTCTTAGAGGCTTCTCTT R:TGTAGCTTCCCACAGAGTTAGAG	149 bp
CD1a	F:GAGTTGTTTCGTCAGTTTCCATAG R:GGAGGCCCTTGGAGTTATCATT	452 bp
CD83	F:CTCTACTGGGCTGTTACCTTGTT R:GAGGAGTTCACACAGAAGACCAT	138 bp
CD86	F:GCCTGAGTGAGCTGGTAGTATTT R:TGTGAAGTCGTAGAGTCCAGTTG	150 bp
β-actin(H)	F:ACATCCGCAAAGACCTGTAC R:GCCATGCCAATCTCATCTTG	346 bp
β-actin(M)	F:CTTTGCAGCTCCTTCGTTG R:TGGTAACAATGCCATGTTCA	278 bp

Immunocytochemical staining. The cells were inoculated to the culture plate at the concentration of 2 × 10^4^/ml for growing on the slide until the cells grew to a relatively uniform concentration; the culture medium was taken out and fixed by ice acetone for 15 min after washing by PBS, followed by 0.5% H_2_O_2_ incubation for 15 min, blocking serum incubation for 20 min, primary antibody incubation at 4 °C overnight, secondary antibody working solution incubation at 37 °C for 30 min, DAB coloring, hematoxylin counterstaining, ethanol decolorizing and mounting with gum.

### SU3-ihDCTC chromosome karyotype analysis

SU3-ihDCTC cells and DCs were submitted for chromosome karyotype analysis by g-banding, followed by addition of colchicine at the final (mass) concentration of 10μg/ml and action at 37 °C for 4 h. After that, the cells were collected and processed by Giemsa staining after hypotonic treatment, fixation and slide dropping. Cell division phase was automatically searched by the platform microscope on chromosomal analyzer.

### SU3-ihDCTC in vivo tumorigenic experiment

SU3-ihDCTC (1 × 10^6^ cells/100 μl) in the logarithmic growth period was inoculated subcutaneously on the right back of 10 non-fluorescent nude mice to observe the tumor induction rate and pathological features. At 21 days after inoculation, 10 mice were anesthetized by intraperitoneal injection of 10% chloral hydrate (200 mg/kg).and were sacrificed by breaking the neck.

### Detection of cell proliferation via CCK-8 assay

Both ihDCTC and SU3 cells were cultured in DMEM high glucose medium containing 10% fetal bovine serum and placed in a 5% CO_2_ incubator at 37 °C. The cells in the logarithmic growth period were inoculated to the 96-well plate (3000 cells/well) with 6 duplicate wells for each group; after culture in the 5% CO_2_ incubator at 37 °C for 24 h, media with different concentrations of Res and Cis were submitted for this procedure and cultured for 24 h, 48 h and 72 h. After that, CCK-8 reagent was added to each well in the dark, followed by culture in the incubator for 2 h and detection of OD value at 450 nm using the microplate reader. The cell survival rate and the half maximal inhibitory concentration (IC_50_ value) of two cell lines for two drugs were calculated. SPSS 22 software was adopted to analyze the OD value of two cell lines treated by Res and Cis, and GraphPad Prism 5 was used for plotting to demonstrate the differences between groups.

### Cell cycle detection using flow cytometer

After trypsin digestion, the ihDCTC cells in the logarithmic growth period was centrifuged at 1000 rpm for 5 min; the supernatant was discarded before adding the medium; after being measured by counting chamber, the solution was diluted into single cell suspension with 10^6^ cells and then tiled in a 6-well plate. The plate was placed in the 5% CO_2_ incubator at 37 °C for 24 h of adherent growth, followed by replacing by media with different concentrations of Res and Cis, where the control group was simultaneously set. After incubation for 24 h, trypsin digestion and centrifugation, the cells were washed by PBS twice before addition of 70% ice ethanol and placement at 4 °C overnight, followed by action by RNaes at 37 °C for 1 h, addition of PI and staining at 4 °C for 1 h. at last, cell cycle was detected by flow cytometer.

### Apoptosis detection using flow cytometer and Hoechst 33,342 staining

#### Apoptosis detection using Annexin-V method

Two cell lines were treated with 10, 50 and 100 μM Res and 5, 10 and 50 μM Cis for 48 h, respectively. The cells were stained by Annexin V-FITC and PI respectively according to the manufacturer’s instructions, and flow cytometer was used to analyze the fluorescence intensity of FITC and PI, thereby quantitatively detecting the apoptosis status of different groups.

#### Hoechst vital cell staining

ihDCTC and SU3 were treated with 100 μM Res and 10 μM Cis respectively for 48 h; after removing the medium, Hoechst33342 was diluted by PBS subject to the ratio of 1:2 and then added to the cells for 20 min of staining. With the staining solution removed, the cells were washed by PBS twice and then photographed under the purple exciting light for counting.

#### Detection of protein expression via western bolt

Cells from each treatment group or tumor tissues from each group of the nude mouse subcutaneous xenograft model were collected and placed in a 1.5 mL EP tube, followed by addition of appropriate amount of lysate for thorough lysis and ultrasonic testing in the ice bath using ultrasonic processor; after centrifugation at 12000 rpm for 15 min at 4 °C, the supernatant was extracted and stored at − 20 °C for subsequent use. The concentration of proteins extracted above was quantified using BCA Protein Quantification Kit; 5× loading buffer was added to the sample, followed by boiling at 100 °C for 5 min to denature the proteins. After processing by SDS-PAGE, the proteins were transferred to the NC membrane using protein transfer device and then blocked by 5% skimmed milk powder for 1 h. After addition of IL-6, p-STAT3 and NF-κB antibodies and incubation overnight at 4 °C, the proteins were washed by TBST three times before 1 h of fluorescent secondary antibody incubation and another three times of TBST washing; Western Blot imaging software Odyssey analyzer was used to detect and produce images.

#### Animal experiment

ihDCTC thawed from liquid nitrogen was placed in DMEM high glucose medium containing 10% fetal bovine serum and cultured in 5% CO_2_ incubator to the vigorous growth period; the cells were then inoculated to 21 nude mice (age, about 6 weeks; weight, about 22 g) at the concentration of 1 × 10^6^ cells/animal. When the tumor grew to the extent that vernier caliper could be used for measuring, the animals were divided into A) control group (*n* = 4); B) ihDCTC Res treatment group (*n* = 5); C) ihDCTC Cis treatment group (n = 4); and D) ihDCTC Res + Cis treatment group (n = 4), where the Res dose was 12.5 mg/Kg and intraperitoneally injected daily for three consecutive weeks, while Cis was injected every other day for three consecutive weeks at the dose of 2 mg/kg. The body weight and tumor size of modeled mice were measured every 3 days. Tumor volume was calculated by formula a × b^2^÷2, where a represented tumor long diameter, while b represented tumor short diameter, followed by plotting the proliferation curve. Blood was collected from the caudal vein weekly and submitted for blood routine. At the end of the experiment, as previously described,mice were anesthetized by intraperitoneal injection of 10% chloral hydrate (200 mg/kg).blood was sampled from the eyeball with their liver and kidney function examined,and the node mice was sacrificed.the tumor tissue was removed and weighed in the aseptic condition, and part of the tissue was cut into pieces to prepare cell suspension and perform conventional cell culture. After that, paraffin section and immunohistochemical staining were conducted to analyze the traditional pathological and molecular pathological features of xenografts.

#### Statistical methodology

The experiment was duplicated at least 3 times. GraphPad Prism 5 software was used for imaging analysis; one-way ANOVA and Student’s t-test were employed for data statistical processing, where the data were represented by mean ± standard deviation (x ± SD). *P* < 0.05 was defined as statistical significance.

## Results

### Identification of malignant transformation of bone marrow derived dendritic cells (BMDCs) induced by SU3-RFP using fluorescent tracer

**Fig. 1:**
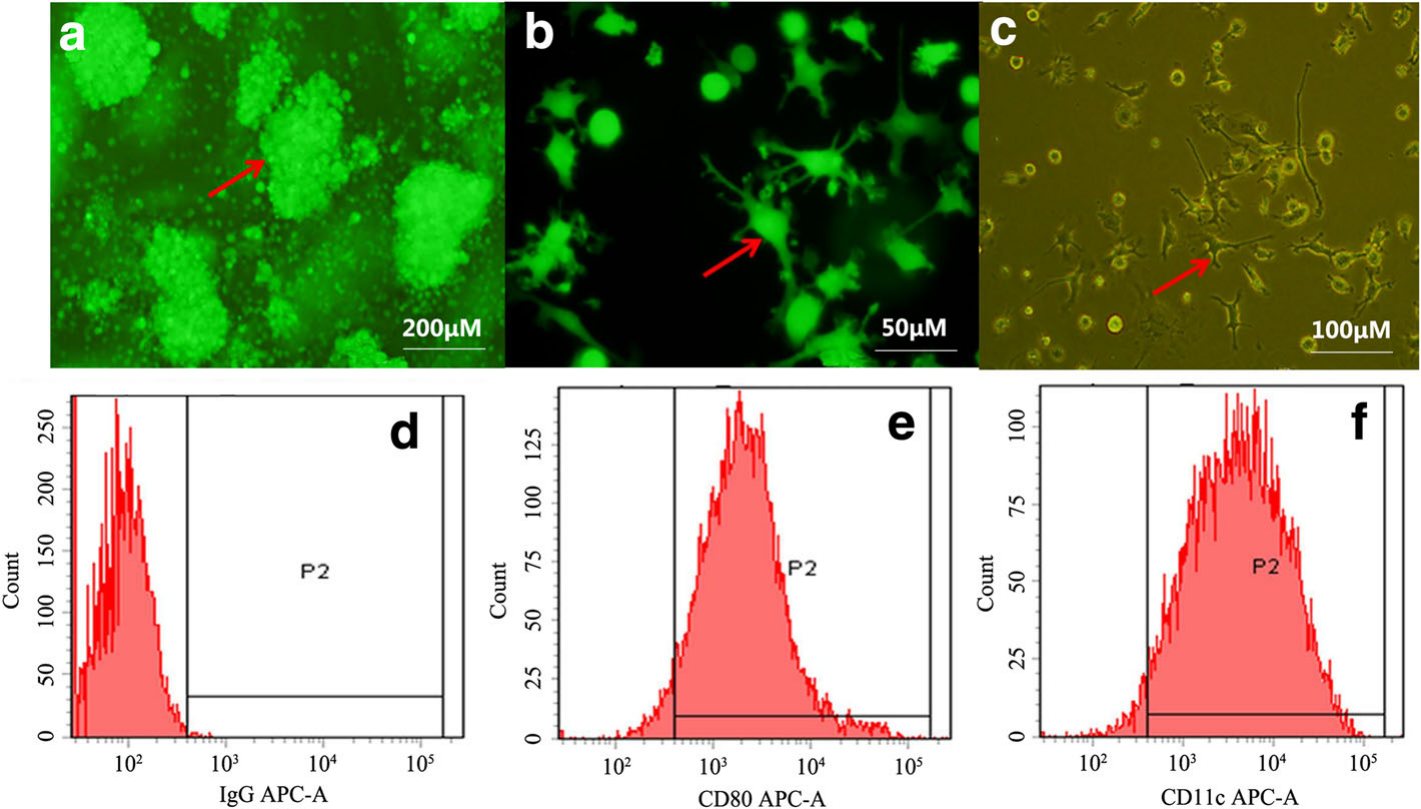
Bone marrow derived dendritic cells cultured in vitro: **a** cell colony. **b** Hemiparietal cell observed by Fluorescence microscope. **c** Hemiparietal cell observed by inverted microscope. d-f CD80,CD11c were detected by Flow cytometer. **d** IgG negative control **e** the percentage of CD80 positive cell is 78.4% **e** the percentage of CD11c positive cell is 73.2%

**Fig. 2:**
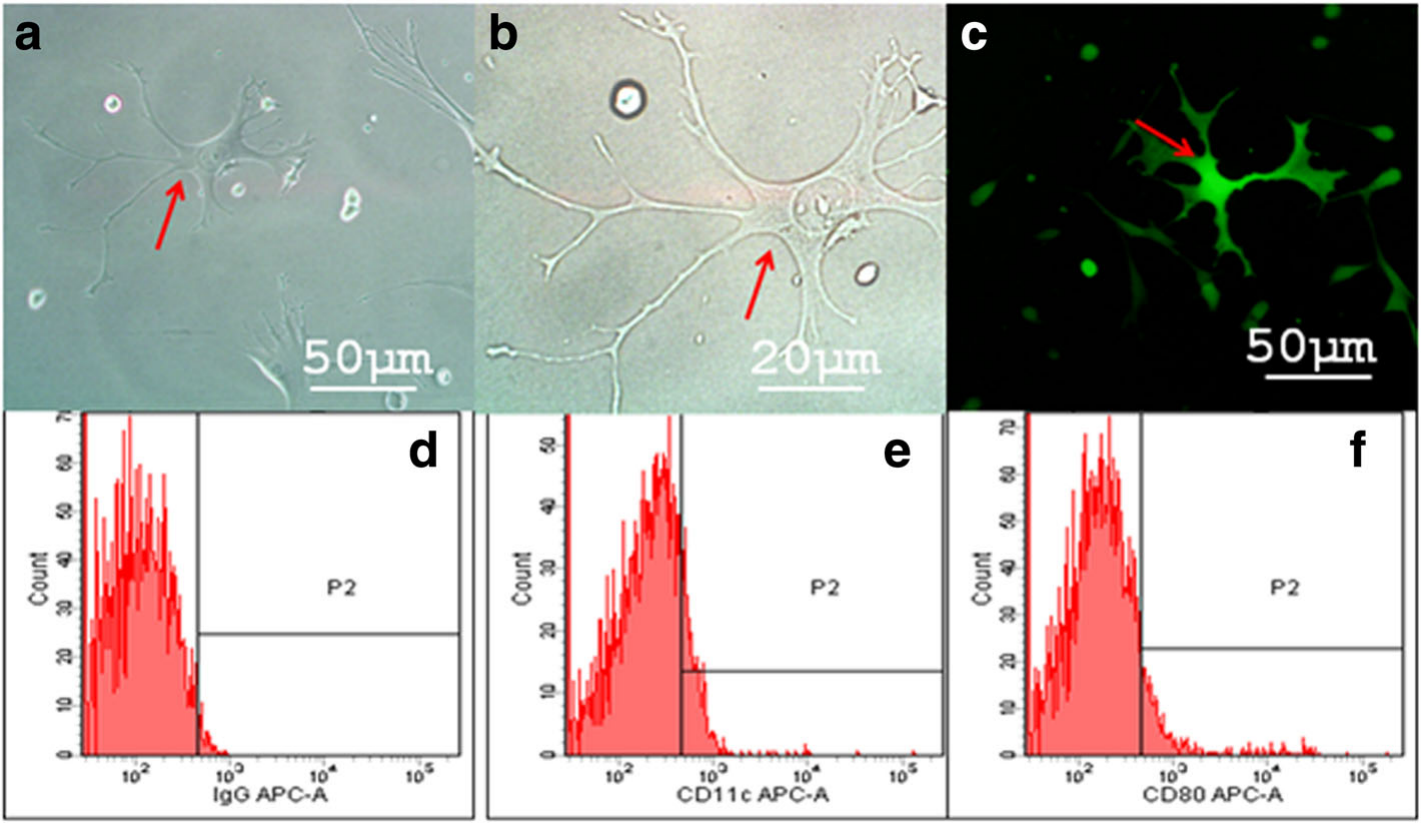
Acquire Common expression of EGFP and RFP double positive cells in co-cultured system: **a**, **b** obversed by the white light microscope **c** obversed by fluorescence microscope;cell morphological characteristics conform to the characteristics of dendritic cells(arrows in fig **a**, **b**); (**d**-**f**) The expression of CD80、CD11c on EGFP and RFP double positive cells were detected by Flow cytometer. **d** IgG negative control; (**e**) the percentage of CD80 positive cell is 47.4% (**e**) the percentage of CD11c positive cell is 42.9%

The established mouse DC cell line was cultured for 8 days to collect the suspended cells, when the microscopic findings showed many ambient burr-like and “hedgehog-shaped” processes, while some cells might presented adherent growth in the “dendritic” form. All these cells emitted green fluorescence under inverted fluorescence microscope (Fig. 1a-c). The detection of molecular markers CD80 and CD11c on the surface of these DCs via flow cytometer found that the cells positively expressing DC marker proteins accounted for approximate 80% (Fig. 1d-f). The obtained normal DCs were directly mixed and co-cultured with SU3-RFP cells, as the former could highly express EGFP while the latter could highly express RFP, thus easily distinguishing them under inverted fluorescence microscope. In the early phase, the SU3-RFP cells was only 1/10 of the concentration of normal DCs, which led to dominant proportion of green cells rather than red cells. However, the red cells continued to grow with the extension of incubation. Duplications were conducted until fluorescence microscope showed occasional green cell colonies and yellow cell colonies among the red cell population. After culture and proliferation, the green cell colonies were submitted for monoclonal treatment, and the obtained green cells maintained the typical “dendritic” form. Therefore, SU3-ihDCTC reported in our study was derived from DCs expressing only EGFP, and cells expressing molecular markers CD80 and CD11c on their surface could be approximate 50% as detected by flow cytometer (Fig. 2).

### Molecular identification and tumorigenesis experiment study of malignantly transformed SU3-ihDCTC

**Fig. 3:**
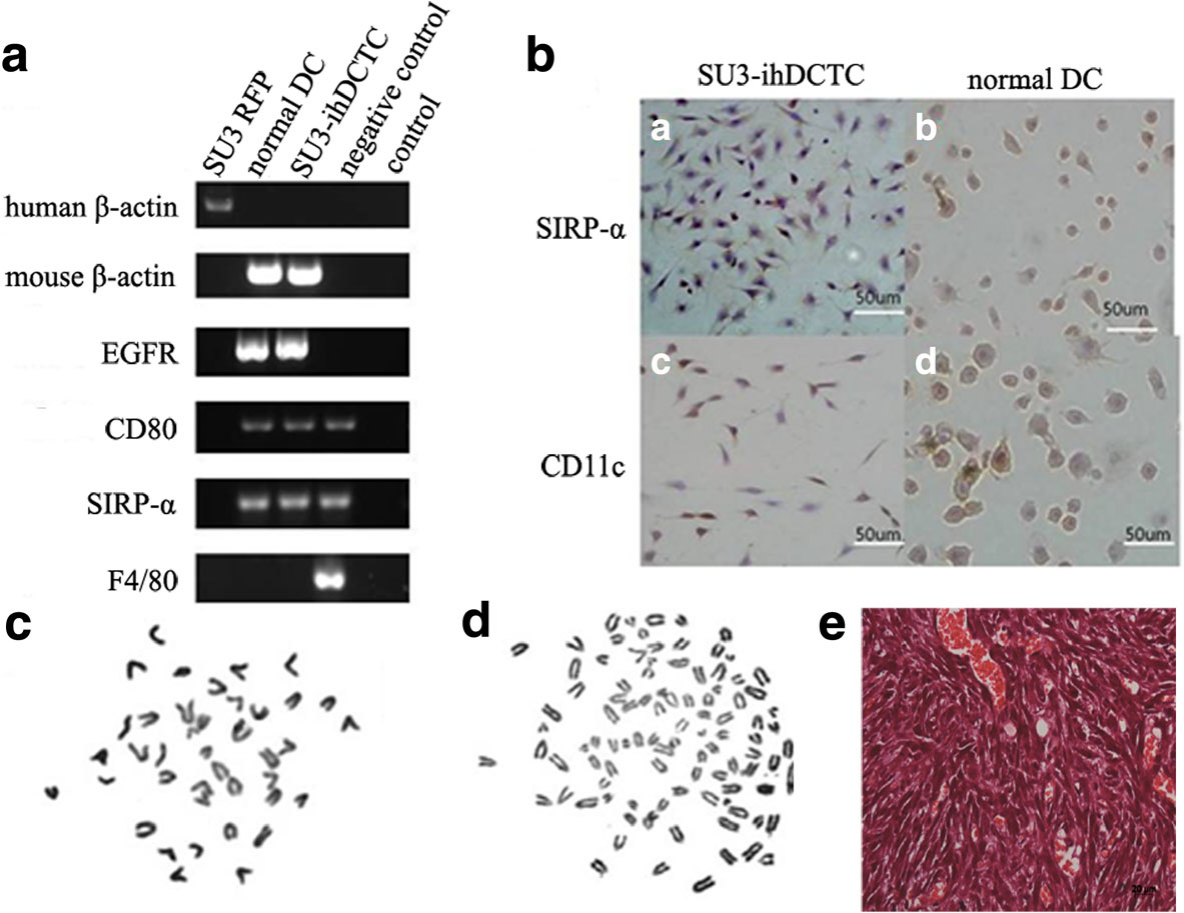
The of Molecular and chromosome phenotype of SU3-ihDCTC and normal DCs: (**A**) The expression of normal DCs expressing specific molecular markers and the host expressing specific molecular markers were detected by RT-PCR in normal DCs、SU3-RFP、SU3-ihDCTC. SU3-ihDCTC express mouse β-actin, EGFP, CD80 and SIRP-α but would not express F4/80 just like the case of normal DCs; (**B**)The expression of CD11c and SIRP-α were detected in both SU3-ihDCTC and normal DCs via immunocytochemical staining:both SU3-ihDCTC and normal DCs were expressing CD11c and SIRP-α. (**C**) The chromosome karyotype of mouse (**D**) The chromosome karyotype of SU3-ihDCTC. (**E**) The xenografted tumor pathological hematoxylin-eosin (HE) staining

Detection of CD80, SIRP-α, F4/80, β-actin and EGFP via RT-PCR indicated that SU3-ihDCTC could express mouse β-actin, EGFP, CD80 and SIRP-α but would not express F4/80 just like the case of normal DCs (Fig. 3A). Detection of CD11c and SIRP-α via immunocytochemical staining showed that SU3-ihDCTC, similar with normal DCs, expressed CD11c and SIRP-α but did not express F4/80 (Fig. 3B). Flow cytometry analysis revealed that the expression ratio of CD80 and CD11c in SU3-ihDCTC cells was 47.4 and 42.9% respectively (Fig. 2d-e). Routine chromosome karyotype analysis suggested that SU3-ihDCTC was a heteroploid containing about 90 chromosomes, whereas the normal DCs were normal telocentric diploid (Fig. 3C-D). SU3-ihDCTC and normal DCs were subcutaneously inoculated to 5 non-fluorescent nude mice respectively, and at 21 days after inoculation, SU3-ihDCTC induced tumor in all 5 mice with the average tumor diameter of about 10 mm. The traditional pathological hematoxylin-eosin (HE) staining showed dense round and fusiform tumor cells, which presented deeply stained large nuclei, less cytoplasm and common nuclear division. Tumor cells invaded to the intermuscular space, subcutaneous fat and hair follicles. With varying inner diameters, most tumors developed vessels with red blood cells visible in their lumen; however, no red blood cells were observed outside the lumen, suggesting limited hemorrhage and necrosis, which might be associated with the insufficient progress of obtained xenografts to middle and advanced stages (Fig. 3E).

### Sensitivity of ihDCTC and SU3 to res and Cis

**Fig. 4:**
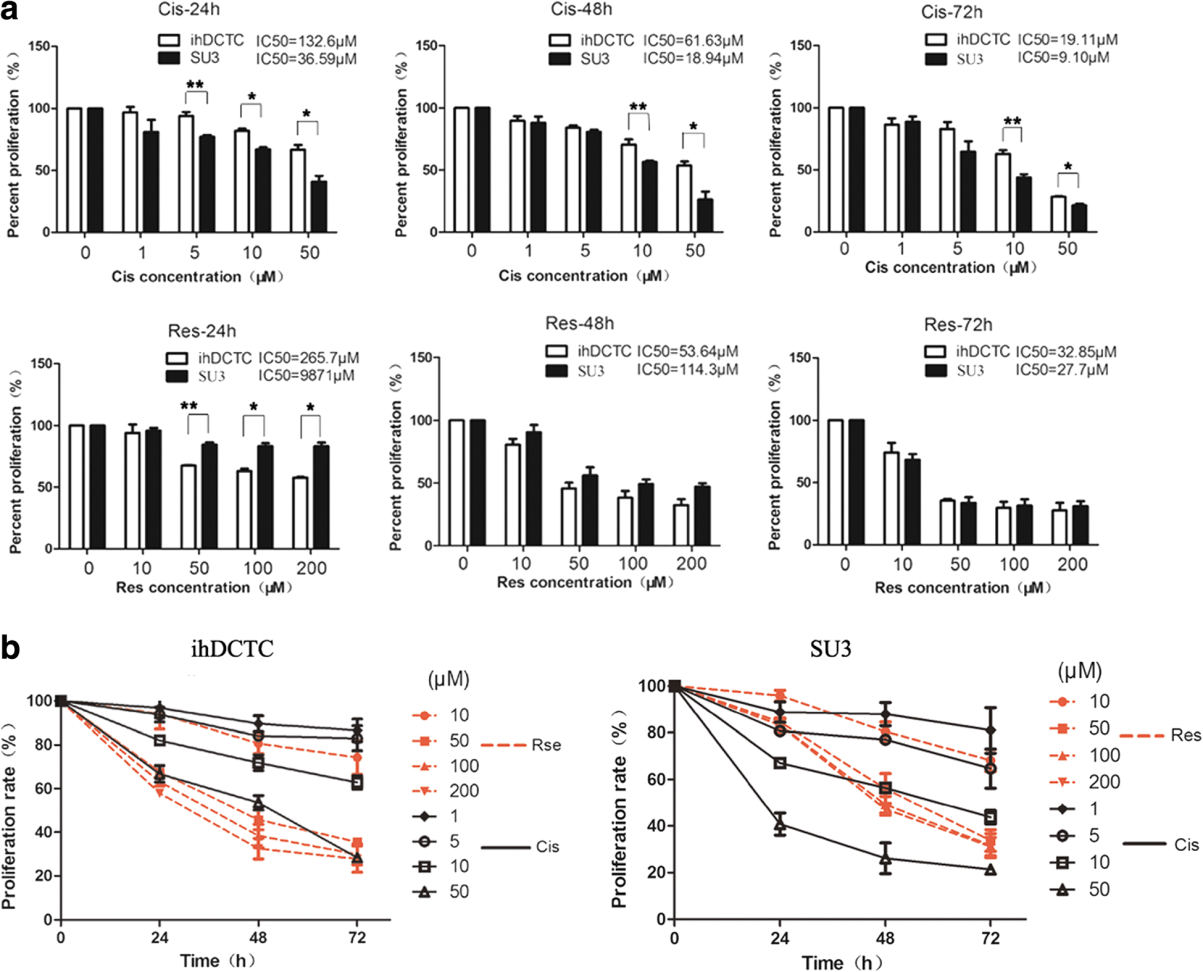
The sensitivity of ihDCTC and SU3 to resveratrol (Res) and cisplatin (Cis).ihDCTC is more resistant to traditional chemotherapeutic drug Cis than SU3. survival rate of different concentration gradient’s Cis and Res deal with ihDCTC and SU3 for 24 h、48 h、72 h (**a**). Line chart of the same data is (**b**). Compared with different cells treated with the same drug, *P < 0.05, **P < 0.01

In order to detect the respective effect of Res and Cis on the proliferation of two cell lines, the proliferative capacity of drug-treated cells were determined using CCK-8 assay, followed by plotting the survival rate histogram and survival rate curve of different concentrations according to the experimental results. The results suggested that Cis could significantly better inhibit SU3 proliferation than ihDCTC, and the difference remained statistically significant both after action for 24/48/72 h and at the concentrations of 5, 10 and 50 μM (*P* = 0.019/0.003/0.013 at 5/10/50 μM after 24 h; *P* = 0.039/0.034 at 10/50 μM after 48 h; *P* = 0.011/0.01 at 10/50 μM after 72 h) (Fig. 4a), demonstrating that ihDCTC cells appeared to be more resistant to Cis than SU3. In addition, the inhibitory effect of Res on the proliferation of SU3 cells was not obvious, which was consistent with the report by Castino et al. about glioma cells U87MG [14]. However, compared with Cis, Res exhibited a significant inhibitory effect on ihDCTC cells after 24 h of action and the difference was statistically significant (P = 0.011/0.008/0.015 at 50/100/200 μM respectively). No significant differences were observed in the inhibitory effect of Res on two cell lines after 48 h and 72 h of action. Nevertheless, our results found a significant difference in the inhibitory effect on ihDCTC proliferation between different concentration gradients of Res and Cis, suggesting that Res could significantly inhibit the proliferation of ihDCTC (Fig. 4b).

### Res could block ihDCTC cells from the S phase to the G_2_-M phase

**Fig. 5:**
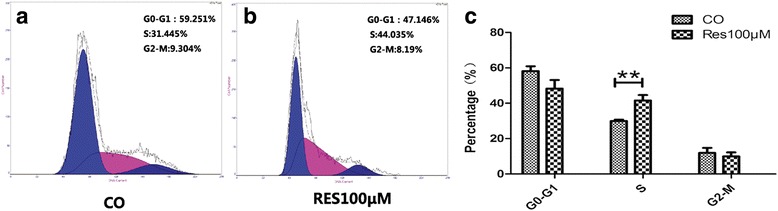
Res block ihDCTC cells from the S phase into the G2-M phase. Flow cytometry was used to detect the changes of ihDCTC cells in the cycle after 100 μM Res 48 h (**b**). compared with control groups (**a**). The Related data statistical histogram is (**c**) **P < 0.01

Res could significantly inhibit the proliferative capacity of ihDCTC cells. According to Fig. 1, the 24-h survival rate of ihDCTC treated by 10, 50, 100 and 200 μM Res was 94.09 ± 9.74%, 67.58 ± 0.54%, 63.14 ± 2.88% and 57.87 ± 0.87% respectively. In order to investigate whether the inhibitory effect was achieved by regulating the cell cycle, the drug concentration should be enough to arrest the cell cycle while inadequate to generate cytotoxic effects as a result of over dose. In consequence, the concentration of 100 μM, which was close to the IC_50_ value, was adopted to detect the changes in different phases of ihDCTC cells after 24 h of Res action using flow cytometer. The results demonstrated obvious changes in G_0_-G_1_, S and G_2_-M phase ratios (%) in both the administration group and the control group (Fig. 5a and b), where the S phase ratio in the administration group showed a statistically significant rise (*P* = 0.0107) (Fig. 5c), indicating that the inhibitory effect of Res on the proliferation of ihDCTC cells was associated with S phase arrest and subsequent impact on cell DNA synthesis.

### Effects of res and Cis on the apoptosis of ihDCTC cells

It has been reported that both Res and Cis can induce tumor cell apoptosis and thus realize tumor inhibition [15, 16]. We hypothesized that the different inhibitory effect of Res and Cis on ihDCTC proliferation might be associated with the different ihDCTC apoptosis status induced by them. In order to verify this hypothesis, ihDCTC apoptosis status was detected after 48 h of Res and Cis treatment respectively.

**Fig. 6:**
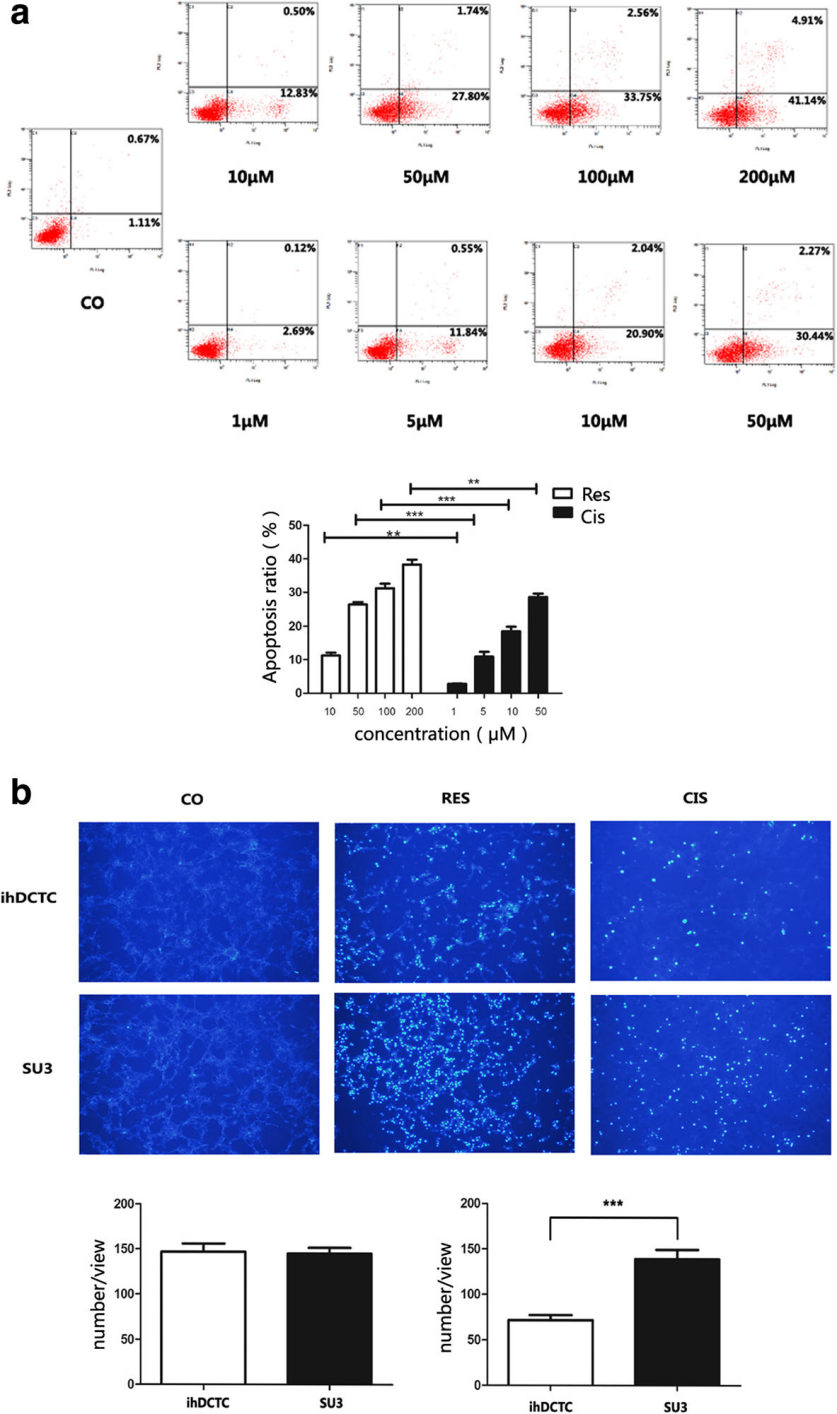
Res have stronger ablity to induce apoptosis of ihDCTC cells then Cis. **a** Flow cytometry was used to detect different concentration gradient’s Res(10、50、100、200 μM)and Cis(1、5、10、50 μM) deal with ihDCTC for 48 h.Left lower quadrant was normal cells, upper right quadrant was late apoptotic cells, early apoptosis cells at the lower right quadrant. **b** 100 μM Res and 10 μM Cis were treated with ihDCTC and SU3 48 h and Hoechest33342 for 30 min respectively. Apoptotic cell nucleus which was stained blue were obversed under the purple excitation light by Fluorescence microscope. Compared with control groups. **P < 0.01

The results showed that varying degrees of apoptosis were observed in all ihDCTC cells treated by different concentrations of Res and Cis. Compared with the Cis treatment group, the Res treatment group exhibited significantly higher apoptosis percentage and followed a dose-dependent tread (Fig. 6a). In addition, ihDCTC and SU3 cells were treated by 100 μM Res and 10 μM Cis respectively for 48 h and then stained using Hoechest33342 for 30 min; according to the observation under the purple excitation light of fluorescence microscope, cells stained blue were apoptotic nuclei. The staining results of Hoechest33342 were also consistent with the foregoing findings (Fig. 6b).

### Evaluation of the effect of res and Cis on ihDCTC Xenografts and its re-cultured cell proliferation

**Fig. 7:**
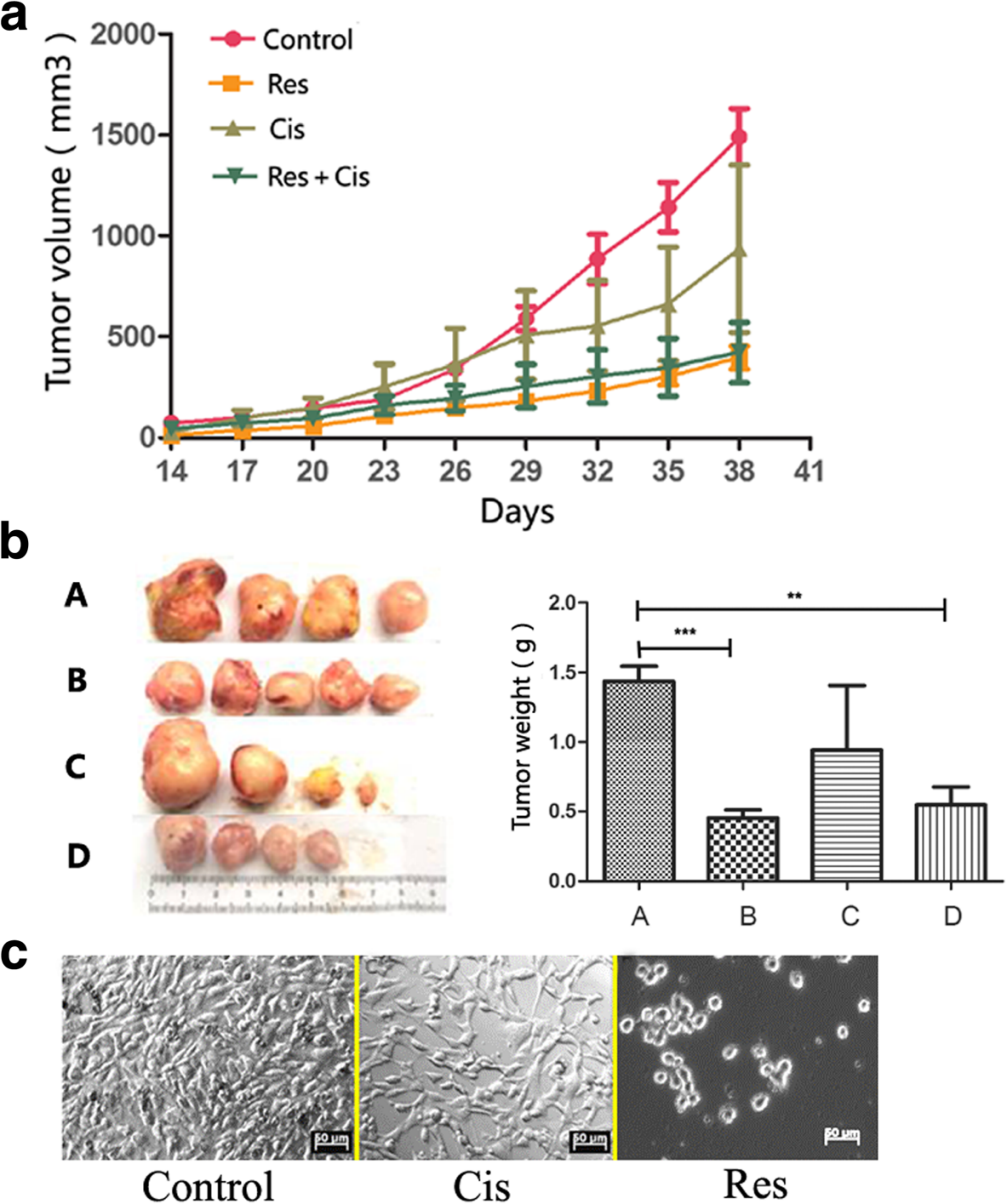
Res can inhibit transplantation tumor of ihDCTC effectively,but Cis is the opposite. **A** In the development of the treatment of the tumor, the tumor volume changes with time. **B** For the end of the experiment,Transplanted tumor photo graph and Transplanted tumor weight Statistical graph (*A* was control group; *B* was group of Res treatment; *C* was group of *C* is treatment; *D* was group of combination treatment) (**C**) For the end of the experiment,Transplanted tumor tissue primary culture for 7 days,Observed by inverted microscope(50 μm). Compared with control groups. **P < 0.01, ***P < 0.001

After ihDCTC incubation, the latency of tumor growth was about 7 days, and animals were randomly divided into the control group (A), Res group (B), Cis group (C) and Res + Cis combined treatment group (D) after 14 day, when the tumor volume had reached about 40mm^3^ and no inter-group statistical differences were reported. With the extension of treatment course, tumor growth was significantly inhibited in the Res group on the 29th day (mm^3^) (175.08 ± 37.25, *P* = 0.016) compared with the control group, while tumors in the ihDCTC combined treatment group and the Cis group failed to be inhibited on the 32nd day (227.89 ± 49.35, P = 0.01) and 38th day (936.375 ± 721.14, *P* = 0.076) respectively (Fig. 7A). On the 38th day when the experiment ended, the mass of tumors was 1.44 ± 0.19 for group A, 0.45 ± 0.12 (*P* = 0.008) for group B, 0.94 ± 0.80 (*P* = 0.164) for group C and 0.68 ± 0.35 (*P* = 0.039) for group D.

Compared with the control group in terms of tumor proliferation curve and tumor mass, the tumor proliferation was significantly inhibited in the Res group while the situation for the Cis group was just the other way around; no statistically significant difference was observed between the combined treatment group and the Res group (Figs. 7A and B). According to the tumor re-culture results, the control group was featured by active cell proliferation and dense arrangement, the Cis group also exhibited active cell growth and less dense arrangement, while the Res group was characterized by few survived cells, and all those survived were small round cells (Fig. 7C).

### Evaluation on the sensitivity of tumor-bearing mice to drug toxicity and treatment

An antitumor drug is not only judged by its inhibitory effect on tumors but also by its toxic and side effect on normal cells. Cis is a widely used broad-spectrum anti-cancer drug, but its toxicity to the organism limits its high-dose application in clinical practice [17]. In order to determine whether the antitumor effect of Cis would cause toxic reaction in the organism, the WBC and body weight data in blood routine reports of tumor-bearing mice were collected during the treatment course to compare the toxicity of two drugs to the organism (Table 2). Significant decrease of WBC was observed in the Cis group only, whereas the decrease in the Res group and Res + Cis group was not obvious. The calculation results according to formula final body weight (FBW) ÷ initial body weight (IBW) have been listed in Table 2, which indicated that the decrease in mouse body weight in the Cis group and the Cis + Res group failed to meet the withdrawal criteria (less than 0.85), whereas the Res group hardly exhibited any toxicity. This suggested that the traditional anticancer drug Cis is featured by the toxicity of bone marrow suppression for nude mice, but the weight loss remained acceptable. However, no significant decrease in WBC and body weight were observed in the Res group, and it has been shown that higher intraperitoneal doses were still tolerable for nude mice [18], suggesting that increased Res dose may more effectively inhibit tumor growth while cause no obvious toxicity.

**Table 2:** weight and white blood cells changes of tumor-burdened mouse before and after treatment

Group	Body weight ($\overline{\mathrm{x}}$±S g)			Peripheral blood (WBC, $\overline{\mathrm{x}}$ ±S,10^6^)		
	IBW	FBW	FBW/IBW	pretreated	first week	second week
A	21.00 ± 2.64	24.28 ± 2.10	1.16	12.38 ± 3.39	12.73 ± 3.48	17.01 ± 8.20
B	21.58 ± 2.34	24.90 ± 1.91	1.15	9.28 ± 0.58	9.70 ± 3.67	8.61 ± 2.23
C	22.88 ± 2.90	20.03 ± 1.10	0.87	17.77 ± 2.51	14.84 ± 2.6	9.82 ± 2.56
D	23.35 ± 0.15	20.67 ± 2.65	0.89	7.26 ± 1.11	7.31 ± 0.21	7.01 ± 1.48

IBW is the initial body weight; FBW is the final body weight; A is control group; B is Res treatment; C is Cis treatment; D is combined treatment

### Study on related molecular mechanism of res effectiveness against ihDCTC

**Fig. 8:**
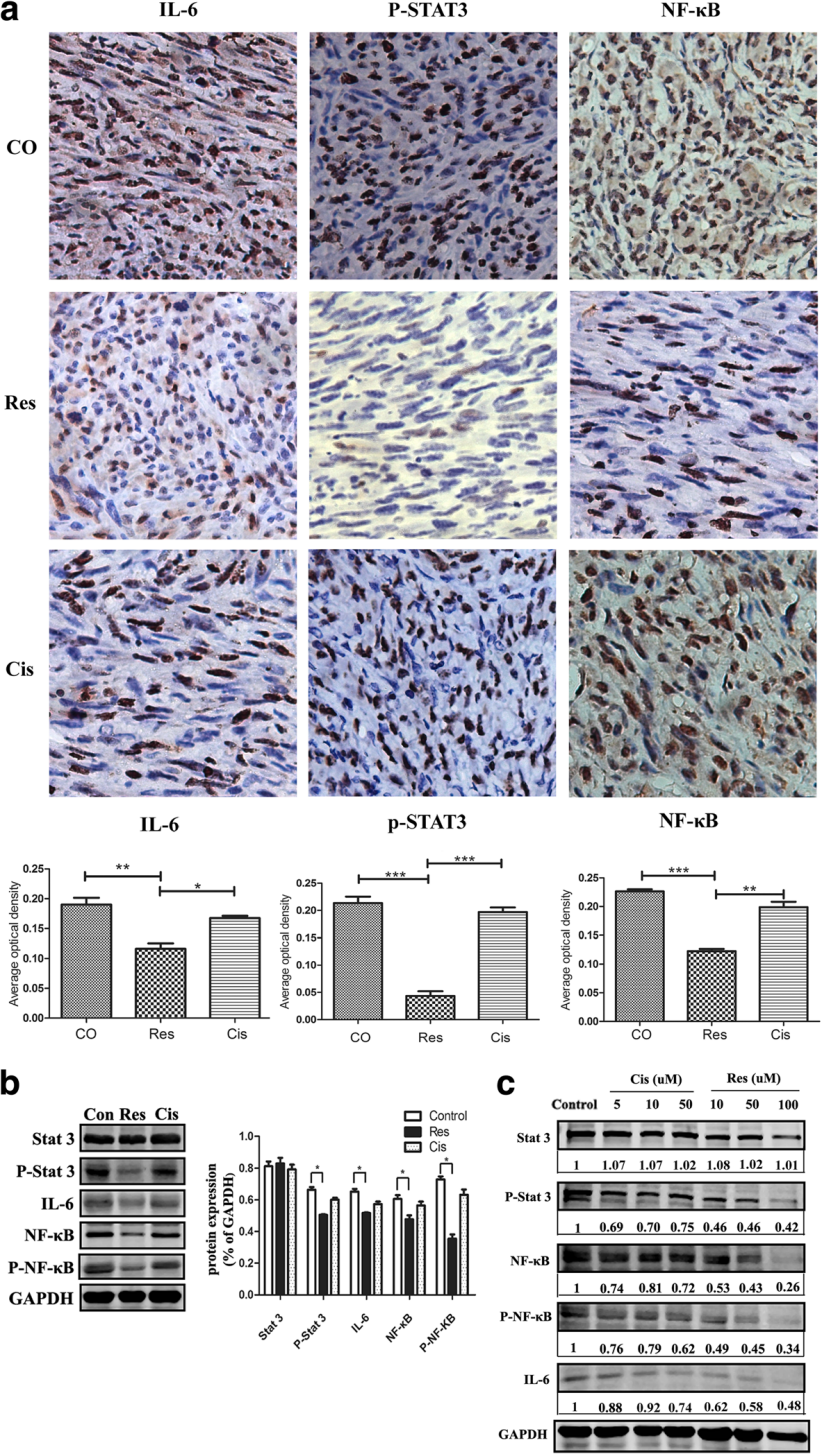
Res can decrease the expression of IL-6, p-STAT3 and NF-κB in transplantation tumor and cells effectively,while the Cis showed a decrease in the expression level, but there was no statistical difference. **a** ihDCTC transplanted tumor tissue immunohistochemistry staining were obversed by light microscope(X400). Brown were Immune complex . **b** Expression of target proteins in ihDCTC transplanted tumor tissue measured by Western blot assay. **c** Expression of target proteins in different concentration gradient’s Cis and Res deal with ihDCTC and SU3 for 48 h,measured by Western blot assay .The number in picture is the ratio of the protein gray to the internal reference GAPDH. * was *P*<0.05

Studies have shown that Res may cause IL-6 changes in cells and further downregulate the expression of p-STAT3 and NF-κB [19]; the development of Cis resistance is also associated with the continuously activated p-STAT3 and NF-κB in cancer cells [20, 21]. In our study, the protein expression levels of IL-6, p-STAT3 and NF-κB were detected via Western Blot in order to explore the different action mechanisms of Res and Cis against ihDCTC cells. The results have been shown in Fig. 8. The immunohistochemical staining results of IL-6, p-STAT3 and NF-κB showed that the expression of these three proteins were significantly reduced in mouse tumor tissue sections of the Res group. According to the Western Bolt results, the control group exhibited higher expression of IL-6, p-STAT3 and NF-κB; the expression of these proteins in the Res group was significantly lower than the control group, and the differences were statistically significant (P_(IL-6)_ = 0.015, P_(p-STAT3)_ = 0.016, P_(NF-Κb)_ = 0.021, *N* = 3); although their expression levels in the Cis group were reduced, the differences showed no statistical significance. These results revealed that the better efficacy of Res in the treatment of ihDCTC tumor-bearing mice than Cis might be associated with the fact that Res could effectively inhibit the expression of IL-6-p-STAT3-NF-κB in xenograft tissue.

## Discussion

SU3-ihDCTC is a malignantly transformed DC strain derived from the bone marrow of nude mice expressing green fluorescent proteins in vitro induced by glioma stem/progenitor cell SU3-RFP, which can highly express EGFP and is featured by immortalization, heteroploid chromosomes and high tumorigenicity in nude mice. According to the mouse DC classification criteria by Stephanie et al. [22] based on cytokine chemokine receptor (XCR1) and signal-regulated protein α (SIRP-α), SU3-ihDCTC belongs to tumor-associated SIRP+DCs and can be further used for studies focusing on nonresolving inflammation cells as a tool cell. In particular, it can be utilized in the research on new antitumor drugs as malignantly transformed cells induced in TME.

Res has been found to exist in 72 plant species distributed in 21 families and 31 genera, including Chinese herbs *Cassia tora*, *Veratrum nigrum* and *Polygonum cuspidatum* [23]. Interestingly, it has also been found in wines [24], which contributes to the research enthusiasm of many scholars. Constant investigations have shown that Res can generate multiple biological effects, such as anti-oxidation, anti-inflammatory and lipid metabolism regulating, and exhibit a wide antagonism against mammalian pathogen-induced infections. Owing to the inhibitory effect on the proliferation of varying tumors at different stages like malignant glioma and melanoma, it has been used for the experimental research focusing on chemoradiotherapy and related target molecules during the past two decades [25, 26]. Studies have suggested that Res can inhibit the growth of glioma U87 cells and promote the apoptosis [27]; it can also permeate the blood brain barrier and be absorbed by brain tissue [14], thereby achieving an effective plasma concentration. However, it has not been reported whether Res can inhibit the proliferation of tumor-associated cells originated from TME, especially the malignantly transformed immunotolerant inflammatory cells induced by tumors, such as ihDCTC cells. Providing that ihDCTC cells are derived from bone marrow DCs and belong to immune inflammatory cells, Res is speculated to be effective from the “anti-inflammatory” perspective, and the results of our experiment appear to be consistent with this theory.

However, the problem is that ihDCTC cells, as malignantly transformed DCs, neither have immunological function nor are immunotolerant. Despite of its nature of cancer cells, the effectiveness to them from the “anti-cancer” point of view remains to be proved compared with those malignant tumors like breast cancer, colon cancer and glioma reported in the literature [28–31]. Considering the relevance research theories about MDSC and NRI in TME, the occurrence and development of almost all malignancies are related with chronic inflammation [1, 2], where those conditions that cannot be cured either by anti-inflammatory or anti-cancer therapies are called NRI. In this regard, only drugs capable of acting against both cancer cells and NRI cells can realize the requirements for cancer treatment. Therefore, in our report, cancer cells were represented by SU3, NRI cells by ihDCTC, developed new drug by Res and traditional anticancer drug by Cis. The results of our treatment experiment indicated that 1) for Cis anticancer action, ihDCTC was more resistant than SU3, and the NRI problem remained unsolved after treatment; 2) for Res, both ihDCTC and SU3 exhibited certain sensitivity, and it may simultaneously solve the “anti-cancer” and “anti-inflammatory” tasks. In order to confirm the special double-edged features of Res for ihDCTC and SU3, its effect on the proliferation and apoptosis of ihDCTC and SU3 was investigated in this paper. The results demonstrated that Res can indeed simultaneously kill both ihDCTC and SU3 cells (Figs. 1 and 4).

Finally, it must be pointed out that the killing mechanism of Res against ihDCTC proves to be extensive, and our study mainly focuses on the process by which Res arrests the cell cycle in proliferation to the DNA synthesis period and induces massive apoptosis. This mechanism has been used in the traditional anti-cancer treatment. As mentioned above, the special point is that ihDCTC cells are related with both MDSC and NRI; the key target molecules for specific treatment should be located in their regulatory network, and their basic approach is to stimulate tumor cells to release proinflammatory factors, chemokines and other factors, which may lead to uncontrolled inflammatory cell proliferation and loss of MDSC immune function. At the same time, these two cell types can secrete cytokine IL-6 to promote tumor growth, while STAT3 and NF-κB expressed in them may be activated by IL-6 and TNF-a in TME and then released to extracellular space; the activated STAT3 and NF-κB pathways can not only promote tumor growth but also facilitate MDSC proliferation and activation. Moreover, IL-6 is bound to STAT3 and NF-κB pathways, which makes STAT3-NF-κB a key regulatory hub. Our findings suggest that after Res treatment, the reduced xenograft mass of ihDCTC-bearing mice is associated with the decrease in the protein expression of IL-6, STAT3 and NF-κB, indicating that this pathway may serve as a key target for the treatment of ihDCTC. However, more critical target molecules are required to be explored, since only the authentic and key target treatment meets the precision medical treatment mode favored by our ages. The tumor should be eventually eliminated, while our study only reported a reduction in tumor mass.

## Conclusions

In conclusions,In vitro co-culture with GSC can induce the malignant transformation of bone marrow derived dendritic cells, on the one hand,which shows higher drug resistance to the traditional chemotherapeutic drug Cis than GSCs, but, on the other hand, appears to be more sensitive to Res than GSCs. In brief,ihDCTC which comes from TME was more resistant than SU3,and Res may be have capable of againsting both cancer cells and NRI cells.which can provide a broader vision not only for the further study on the correlation between TME and tumor drug resistance but also for the exploration of Res anti-cancer value.

## Abbreviations

DCs: Dendritic cells
EGF: Epidermal growth factor
EGFP: Enhanced green fluorescent protein
IC_50_: Half maximal inhibitory concentration
ihDCTC: SU3-induced host Dendritic Cells Transformed Cell
MDSCs: Myeloid-derived suppressor cells
NRI: Nonresolving inflammation
RFP: Red fluorescence protein
rmGM-CSF: Recombinant mouse granulocyte-macrophage colony-stimulating factor
rmIL-4: Recombinant mouse interleukin 4
SIRP-α: Rabbit anti-mouse α signal protein
TME: Tumor microenvironment

## References

[1] Marvel D, Gabrilovich DI (2015). Myeloid·derived suppressor cells in the tumor mieroenvironment: expect the unexpected. J Clin Invest.

[2] Novak ML, Thorp EB (2013). Shedding light on impaired efferocytosis and nonresolving inflammation. Circ Res.

[3] Wu J, Wang D, Dai X (2014). Malignant transformation of glioma stromal cells induced by glioma stem cells heterotopicallyinoculated in host liver. Zhonghua Yi Xue Za Zhi.

[4] Chen YM, Fei XF, Wang AD, Dai XL (2013). Host glial cell canceration induced by glioma stem cells in GFP/RFP dual fluorescence orthotopicgliomamodels in nude mice. Zhonghua Zhong Liu Za Zhi.

[5] Yu T, Yang G, Hou Y (2017). Cytoplasmic GPER translocation in cancer-associated fibroblasts mediates cAMP/PKA/CREB/glycolytic axis to confer tumor cells with. Multidrug resistance. Oncogene.

[6] Hanahan D, Coussens LM (2012). Accessories to the crime: functions of cells recruited to the tumor microenvironment. Cancer Cell.

[7] Tariq M, Zhang J, Liang G, Ding L, He Q, Yang B. Macrophage polarization: Anti-cancer Strategies to target Tumor-associated Macrophage in Breast cancer. J Cell Biochem. 2017; 10.1002/jcb.25895. 10.1002/jcb.25895 28106295

[8] Wan Y, Fei XF, Wang ZM (2012). Expression of miRNA-125b in the new, highly invasive glioma stem cell and progenitor cell line SU3. Chin J Cancer.

[9] Dong J, Dai X-L, Lu Z-H, Fei X-F (2012). Incubation and application of transgenic green fluorescent nude mice in visualization studies on glioma tissue remodeling. Chin Med J.

[10] Inaba K, Inaba M, Romani N (1992). Generation of large numbers of dendritic cells from mouse bone marrow cultures supplemented with granulocyte/macrophage colony stimulatin factor. J Exp Med.

[11] Saeidi M, Masoud A, Shakiba Y (2013). Immunomodulatory effects of human umbilical cord Wharton’s jelly-derived mesenchymal stem cells on differentiation, maturation and endocytosis of monocyte-derived dendritic cells. Iran J Allergy Asthma Immunol.

[12] Barclay AN, Van den Berg TK (2014). The interaction between signal regulatory protein alpha (SIRPα) and CD47: structure, function, and therapeutic target. Annu Rev Immunol.

[13] Powell AE, Anderson EC, Davies PS (2011). Fusion between intestinal epithelial cells and macrophages in a cancer context results in nuclear reprogramming. Cancer Res.

[14] Castino R, Pucer A, Veneroni R (2011). Resveratrol Reduces the Invasive Growth and Promotes the Acquisition of a Long-Lasting Differentiated Phenotype in Human Glioblastoma. J Agric Food Chem.

[15] Narendra P. Singh, Udai P. Singh, Venkatesh L. Hegde, et al. Resveratrol (trans-3,5,4'-trihydroxystilbene) suppresses EL4 tumor growth by induction of apoptosis involving reciprocal regulation of SIRT1 and NF-κB. Mol Nutr Food Res. 2011;55(8):1207–18. 10.1002/mnfr.201000576. 10.1002/mnfr.201000576 PMC3516994 21520490

[16] Sato H, Wu Y, Kato Y, Liu Q, et al. DEC2 expression antagonizes cisplatin-induced apoptosis in human esophageal squamous cell. Carcinoma Mol Med Rep. 2017; 10.3892/mmr.2017.6571. 10.3892/mmr.2017.6571 PMC5482072 28498447

[17] Robson H, Meyer S, Shalet SM (2002). Platinum agents in the treatment of osteosarcoma: efficacy of cisplatin vs.carboplatin in human osteosarcoma cell lines. Med Pediatr Oncol.

[18] Wang L, Long L, Wang W (2015). Resveratrol, a potential radiation sensitizer for glioma stem cells both in vitro and in vivo. J Pharmacol Sci.

[19] Limagne E, Lançon A, Delmas D (2016). Resveratrol interferes with IL1-β-induced pro-inflammatory paracrine interaction between primary chondrocytes and macrophages. Nutrients.

[20] Huang S, Chen M, Shen Y (2012). Inhibition of activated Stat3 reverses drug resistance to chemotherapeutic agents in gastric cancer cells. Cancer Lett.

[21] Chen F, Qin X, Xu G. Reversal of cisplatin resistance in human gastric cancer cells by a wogoninconjugated Pt(IV) prodrug via attenuating casein kinase 2-mediated nuclear factor-κB pathways. Biochem Pharmacol. 2017; 10.1016/j.bcp.2017.03.004. 10.1016/j.bcp.2017.03.004 28288821

[22] Gurka S, Hartung E, Becker M (2015). Mouse conventional dendritic cells can be universally classified based on the mutually exclusive expression of XCR1 and SIRPα. Front Immunol.

[23] M J, Cai L, Udeani GO (1997). Cancer chemopreventive activity of resveratrol,a natural product derived from grapes. Science.

[24] Wu CF, Yang JY, Wang F (2013). Resveratrol:botanicalorigin,pharmacological activity aIldapplications. Chin J Nat Med.

[25] Menicacci B, Laurenzana A, Chillà A (2017). Chronic Resveratrol Treatment Inhibits MRC5 Fibroblast SASP-Related Protumoral Effects on Melanoma Cells. J Gerontol A Biol Sci Med Sci.

[26] Cilibrasi C, Riva G, Romano G (2017). Resveratrol impairs Glioma stem cells proliferation and motility by modulating the Wnt signaling pathway. PLoS One.

[27] Ryu J, Yoon NA, Seong H, Jeong JY (2015). Resveratrol induces Glioma cell apoptosis through activation of Tristetraprolin. Mol Cells.

[28] Chin Y-T, Hsieh M-T, Yang S-H (2014). Anti-proliferative and gene expression actions of resveratrol in breast cancer cells in vitro. Oncotarget.

[29] Fouad MA, Agha AM, Merzabani MM (2013). Resveratrol inhibits proliferation, angiogenesis and induces apoptosis in colon cancer cells: calorie restriction is the force to the cytotoxicity. Hum Exp Toxicol.

[30] Liu B, Zhou Z, Zhou W (2014). Resveratrol inhibits proliferation in human colorectal carcinoma cells by inducing G1/S-phase cell cycle arrest and apoptosis through caspase/cyclin-CDK pathways. Mol Med Rep.

[31] Chang HT, Chou CT, Chen IL (2013). Mechanisms of resveratrol induced changes in [Ca(2+)]i and cell viability in PC3 human prostate cancer cells. J Recept Signal Transduct Res.

